# Examining the mechanisms linking Dark Triad traits to OCB through LMX and political skill: a comparative study of managerial and non-managerial levels

**DOI:** 10.3389/fpsyg.2026.1676347

**Published:** 2026-02-27

**Authors:** Abira Reizer, Aharon Tziner, Amos Drory

**Affiliations:** 1 Department of Psychology, Ariel University, Ariel, Israel; 2 Tel-Hai College, Upper Galilee, Israel; 3 Peres Academic Center, Rehovot, Israel

**Keywords:** dark triad, LMX, OCB, organizational politics, political skill

## Abstract

**Purpose:**

This study examines the relationship between perceived leaders’ Dark Triad personality traits (Machiavellianism, narcissism, and psychopathy) and followers’ organizational citizenship behavior (OCB). We explore how leader-member exchange (LMX) and perceived political skill mediate these effects. The study provides a comparative analysis across managerial and non-managerial levels, revealing how these dynamics function at different hierarchical positions.

**Methodology:**

Data was collected from two independent samples representing distinct hierarchical levels: non-managerial employees (*n* = 378) and managers (*n* = 340). Participants rated their perceptions of their leaders’ Dark Triad traits, political skill, and the quality of their exchange relationship (LMX), as well as their own OCB. We analyzed data using structural equation modeling (SEM) to test the direct and indirect effects.

**Findings:**

Our findings reveal distinct patterns of association for each of the perceived Dark Triad traits. These patterns highlight the complex ways in which the perception of leaders possessing the dark traits impacts OCB through LMX and perceptions of leaders’ political skill.

**Originality and implications:**

This study contributes novel insights by examining the mechanisms through which perceived leadership Dark Triad traits relate to organizational citizenship behavior (OCB) across organizational levels. By revealing the mediating roles of LMX and perceived political skill, this research enhances our understanding of the dynamics of Dark Triad leadership. The findings provide practical insights for organizations addressing the challenges posed by leaders who possess dark traits.

## Introduction

In recent years, organizations have become increasingly aware of how leadership.

Personality traits shape workplace dynamics (LeBreton et al., 2018; Palmer et al., 2020). While positive leadership traits often receive attention, there is growing interest in understanding how negative or ‘dark’ traits affect employees and overall organizational functioning (Cesinger et al., 2023; Wille et al., 2023). This awareness has led researchers to explore the Dark Triad. The Dark Triad (DT) is characterized as a trio of undesirable personality traits within organizational settings, encompassing psychopathy, narcissism, and Machiavellianism (Paulhus and Williams, 2002). In the field of organizational sciences, researchers are increasingly focusing on the repercussions of maladaptive behaviors associated with DT traits (LeBreton et al., 2018; Wille et al., 2023). Individuals with Dark Triad traits are often drawn to managerial roles, as they are prone to fulfill their ambitions for power, prestige, and status with alacrity (Landay et al., 2019; Palmer et al., 2020). For instance, when dark personality leaders wield power, they use manipulative and exploitative tactics, such as selfish, antisocial behaviors, and bullying in the workplace (Moorman et al., 2025).

This study examines the impact of these dark traits on Organizational Citizenship Behavior (OCB), which encompasses employees’ voluntary, helpful actions beyond their formal job responsibilities. Previous work has focused on the adverse consequences of Dark Triad leadership for employees, including Counterproductive Work Behavior (CWB), diminished job satisfaction, and reduced engagement. However, the impact of destructive leadership in general (for reviews, Bhattacharjee and Sarkar, 2024; Mackey et al., 2021) and, specifically, the leadership Dark Triad (Bhattacharjee and Sarkar, 2024; LeBreton et al., 2018) on employee OCB was less explored.

This manuscript aims to expand previous literature by addressing three objectives. First, we examine the leadership of DT and OCB from the perspective of their followers. This less common perception of the DT is based on the idea of ‘the eye of the beholder’ (Mackey et al., 2021). This perspective highlights that perceptions are not passive reflections of reality but active psychological constructions that guide followers’ responses to their work environment. As Grutterink and Meister (2022) suggested, individuals are highly motivated by how they perceive themselves and how they believe others perceive and treat them. Accordingly, integrating followers’ ratings of leadership traits enables a more accurate understanding of how perceived DT characteristics may translate into workplace behaviors, such as OCB. Notably, previous research indicates that when teachers perceive their leaders as embodying destructive leadership, their willingness to engage in OCB declines (e.g., Karadağ and Dulay, 2021). These effects often operate through psychological mechanisms such as reduced psychological capital (PsyCap), suggesting that employee perceptions shape not only attitudes but also behavioral outcomes. Therefore, examining leadership DT traits from the subordinate’s perspective is essential for understanding how dark leadership is internalized and how it ultimately affects employee behavior. This approach is particularly relevant given the potential for socially aversive traits to be masked or misinterpreted (De Clercq and Pereira, 2024) and responds to growing calls for incorporating observer-based assessments in dark leadership research (Moorman et al., 2025; Szabó et al., 2023).

Our second objective is to expand the current understanding of the antecedents to OCB behaviors by mapping the indirect pathways through which leadership DT traits shape OCB, specifically via interpersonal mechanisms such as leader-member exchange (LMX) and perceived political skill. While the DT traits have been frequently associated with adverse workplace outcomes, recent scholarship emphasizes the need to move beyond direct-effect models and examine the mechanisms through which these traits operate (LeBreton et al., 2018; Wille et al., 2023). As far as leadership narcissism, Machiavellianism, and psychopathy may have distinct and differential relationships with workplace outcomes (Palmer et al., 2020), it is important to examine their unique effects. In this study, we explore how these three leadership dark traits affect OCB via unique mediating paths, specifically through (1) the quality of the relationship between leaders and their followers or (2) through the employees’ perception of their political skill. Political skill is considered a powerful tool for effective leadership (Kimura et al., 2019) and for building social capital (Munyon et al., 2021); however, it may sometimes mask self-serving motives (Ferris et al., 2019). In that context, we examine whether followers perceive their DT leaders as possessing high political skill and how this perception impacts their willingness to express OCB. Notably, however, most previous studies have relied on leaders’ self-ratings of political skill. Yet research suggests that followers’ ratings offer a more accurate lens into leaders’ actual influence and behavior in the workplace (Meurs et al., 2010). Therefore, this study centers on follower-rated perceptions of their leaders’ political skill.

Our final objective is to examine our research model among two independent samples: non-managerial employees and managerial employees. Research suggests that employees and managers often perceive organizational environments differently due to variations in hierarchical roles, access to information, and differing expectations (Bergh et al., 2019; Dunger, 2025; Kassing, 2000). Individuals in different hierarchical statuses will respond differently to LMX (Andersen et al., 2020) and political skill (Ferris et al., 2019), as these may align with their expectations and worldview. Building on the argument made by Karanika-Murray et al. (2015), who emphasized that LMX may differ meaningfully across hierarchical levels, we propose that the mediating roles of both LMX and political skill should be assessed separately among managers and non-managers. This approach acknowledges that leadership dynamics may not be uniform across organizational strata.

## Dark Triad traits in leadership

Over the past two decades, researchers have shown growing interest in the Dark Triad, narcissism, Machiavellianism, and psychopathy, and how these traits play out in organizational settings (see LeBreton et al., 2018, for a review). While this area of study continues to gain momentum, our understanding of how these darker sides of personality shape leadership and impact workplace outcomes remains somewhat limited, conceptually complex, and at times unclear (Cesinger et al., 2023; Palmer et al., 2020).

### Machiavellianism

Machiavellianism is a social strategy centered on personal gain. It involves distrust of others, a desire for control and status, and the use of manipulation to navigate social situations for personal advantage (Dahling et al., 2009). Researchers have identified four key aspects of Machiavellianism: distrust of others, desire for control, status aspiration, and amoral manipulation (Dahling et al., 2009; Greenbaum et al., 2017). Scholars recognize manipulation as a central feature, often linked to lower empathy and self-serving goals, which can lead to unethical behaviors such as lying and exploitation (LeBreton et al., 2018). To gain power and control, Machiavellians often rely on impression management, adapt easily to social contexts, and use long-term strategies to achieve self-serving objectives (Bueno-de la Fuente et al., 2025; Jones and Paulhus, 2010). They may initially appear friendly or charming. However, individuals with high Machiavellian traits can display hostility if their objectives are threatened, using tactics such as deception, criticism, gossip, bullying and sabotage (Cohen, 2018; O’Boyle et al., 2013). In some cases, Machiavellians may engage in theft and the misuse of resources (De Clercq et al., 2019).

As leaders, Machiavellians tend to express abusive supervision, characterized by mistreatment of subordinates (Drory and Gluskinos, 1980; Mackey et al., 2017; Wisse and Sleebos, 2016). However, not all Machiavellian leaders exhibit these behaviors. Some individuals may suppress their negative traits to conform to social norms that serve their interests. Furthermore, Machiavellians can build trust and respect while covertly exploiting situations to their personal advantage. For instance, they may present themselves as confident and assertive leaders, allowing them to attain and retain leadership roles (Cesinger et al., 2023; Palmer et al., 2020).

### Narcissism

Narcissism is characterized by an inflated self-image, a constant need for admiration, a tendency to take advantage of others, and a lack of empathy, often resulting in insensitive behavior (LeBreton et al., 2018). To protect their self-image, narcissists may ignore criticism and overlook long-term consequences of their actions, which makes it harder for them to learn from mistakes (Grijalva et al., 2020). This personality trait, characterized by vanity and excessive self-love, is commonly found in top management (Palmer et al., 2020). Narcissistic leaders may initially be viewed as capable managers, whose traits are positively correlated with occupational success, characterized by respectable leadership roles, hierarchical advancement, and prestigious jobs (Nuzulia and Why, 2020). However, narcissistic leaders can have harmful effects. These leaders may exhibit self-centered behaviors that tend to reduce employee motivation (Liu et al., 2017). Over time, employees exposed to such leadership may suffer from decreased well-being (Tokarev et al., 2017), lower self-esteem (Fehn and Schütz, 2021), depleted personal resources (Moorman et al., 2025), and higher turnover rates (Nevicka et al., 2018).

### Psychopathy

Psychopathy is characterized by emotional coldness and difficulty forming genuine connections with others. It includes a lack of empathy and remorse, impulsive actions, antisocial behavior, and manipulative tendencies (LeBreton et al., 2018). People with psychopathic tendencies may use aggression and bullying to maintain their power and suppress threats while projecting a charming and positive image to their superiors (Fritzon et al., 2019). Impression management is essential for psychopaths who switch between “Dr. Jekyll and Mr. Hyde” as it allows them to hide their superficial ruthlessness behind a mask of charm proficiently (O’Boyle et al., 2013). Motivated by a strong desire for power, psychopaths lie, exploit others, and act without regard for the consequences on others (Babiak and Hare, 2019).

In today’s business environment, marked by rapid changes and competition, individuals with psychopathic traits can succeed and sometimes rise through the ranks quickly, while leaving a trail of damage behind (Hurst et al., 2019; Landay et al., 2019). These individuals may thrive in jobs that focus more on tasks than people (O’Boyle et al., 2013; Rauthmann and Kolar, 2013). However, psychopaths’ use of harsh tactics and their disregard for responsibility can cause negative consequences. Examples include increased workplace interpersonal aggression, public criticism, bullying, rudeness, unsafe working conditions, and even crossing legal and ethical lines (Mathieu et al., 2014). Research also indicates that employees working under such leaders often perform worse, feel less satisfied at work, experience more stress (Mathieu et al., 2014), and are more likely to exhibit poor behavior themselves (Schyns and Schilling, 2013). Overall, psychopathic leaders usually cause long-term harm to both employees and the workplace (Nuzulia and Why, 2020).

## Dark Triad and organizational citizenship behavior

The concept of OCB includes individual voluntary actions that exceed formal job requirements and are not recognized by the organization’s performance management procedures. These behaviors are not part of an employee’s job description but are instead actions taken by personal choice and initiative that support how the organization operates. Such actions benefit the organization by contributing to its environment and functioning requirements (Organ et al., 2006; Rotundo and Sackett, 2002). OCBs are valuable for organizations because they enhance the overall effectiveness of organizational operations.

Studies have established a connection between OCB and significant outcomes, including individual and unit-level performance (Podsakoff et al., 2009). Various types of organizational behaviors can be defined as OCBs, for example: altruistic help to others who have high workloads, preventing conflicts at the workplace, respecting others’ rights, following rules and regulations even if not directly monitored by supervisors, and not complaining about trivial issues (Srivastava and Saldanha, 2008). What ties all of these behaviors together is their contribution to smoother operations, such as better resource use, planning, and problem-solving (e.g., Organ et al., 2006; Podsakoff et al., 2009). These behaviors may also express loyalty and identification with organizational goals (Chahal and Mehta, 2010).

More specifically, OCB can be examined in two main ways: as a single overall concept (Chen et al., 1998; Miles et al., 2002), or by looking at its different aspects (e.g., Organ, 1988). Organ (1988) proposed a five-part model of OCB that includes altruism, courtesy, consciousness, civic virtue, and sportsmanship. In this study, we follow Spector et al.’s (2010) broader view of OCB and treat it as one overall concept, rather than breaking it down into separate parts.

Research on how leadership traits in the Dark Triad affect outcomes like OCB is still limited in scope, and often unclear or inconsistent (LeBreton et al., 2018; Palmer et al., 2020). We suggest that it is necessary to take a more specific approach that looks at each DT trait individually, using trait-specific models. For example, Machiavellian leaders may lower employee OCB because these leaders often seek control and status, and may act with aggressiveness (Dahling et al., 2009; Wisse and Sleebos, 2016). In fact, studies show a strong link between Machiavellian leadership and abusive supervision (Wisse and Sleebos, 2016), which is likely to lead to reduced OCB among employees (Den Breejen-de Hooge et al., 2021; Mackey et al., 2021).

Narcissistic leaders exhibit self-centered behaviors, often undermining employees’ commitment to OCBs. When leaders focus on their own needs and ignore those of their team, employees may feel unappreciated and unsupported, leading them to pull back from helping others or going the extra mile (Carnevale et al., 2018; Liu et al., 2017). Furthermore, narcissists may overlook the needs of their subordinates (Braun et al., 2018), intensify employees’ psychological strain (Carnevale et al., 2018), and create feelings of distance or disconnection in the workplace (Ong et al., 2016), behaviors that ultimately hinder employee OCB. Indeed, in a dyadic sample, Wang et al. (2021) observed that leader narcissism had a direct negative effect on employee OCB toward the leader and a negative indirect effect *via* perceived insider status.

The most damaging component of the leadership DT is psychopathy (Moorman et al., 2025). Psychopathy is a strong predictor of OCB in a cross-sectional, self-reported sample (e.g., Szabó et al., 2018). Furthermore, psychopathic leaders often use intimidation, bullying, and aggressive persuasion methods, resulting in reduced OCB (Volmer et al., 2016). These leaders create an atmosphere where employees feel pressured, micromanaged, and undervalued, which reduces their motivation to go beyond the minimum required.

We suggest that employees’ perception of their leaders’ DT traits can decrease their willingness to engage in OCB. Our argument is based on cumulative research on dark leadership and the Conservation of Resources (COR) theory (Hobfoll et al., 2018). According to the theory, employees’ work-related emotions and behaviors are motivated by their tendency to protect their existing resources and minimize the risk of further resource loss in resource-draining situations (Hobfoll, 2001; Hobfoll et al., 2018). The potential for resource depletion, particularly in response to distressing work experiences, drives employees to adopt actions that help them mitigate this depletion and cope with challenges (De Clercq and Pereira, 2024; Hobfoll et al., 2018).

For example, previous research has applied COR theory to demonstrate that abusive supervision is resource-consuming (Kacmar et al., 2013). In such toxic or anxiety-provoking environments, like those shaped by DT leaders, employees may withdraw from job involvement to cope with stress and reduce the emotional toll (Khalid et al., 2024). Furthermore, under abusive supervision, employees may be disinclined to engage in citizenship behaviors that could put their resources at risk (Bhattacharjee and Sarkar, 2024). Hence, we propose the following hypotheses:

*H1*: Dark leadership traits, Machiavellianism, narcissism, and psychopathy decrease employee OCB in both the managerial and non-managerial samples, such that:

*H1a*: Machiavellian leadership is negatively related to employees’ OCB in both the managerial and non-managerial samples.

*H1b*: Narcissistic leadership is negatively related to employees’ OCB in both the managerial and non-managerial samples.

*H1c*: Psychopathic leadership is negatively related to employees’ OCB in both the managerial and non-managerial samples.

## Leader-member-exchange (LMX) mediates the relationship between leadership DT and OCB

Leader-Member Exchange (LMX) theory explains how the quality of the relationship between leaders and employees affects job performance and workplace behaviors. Built upon role theory and social exchange theory, LMX theory posits that leadership style differentiates one employee from another, contingent upon their relationship (Yuan et al., 2023). Employees with high-quality relationships with their managers (high LMX) tend to enjoy better work opportunities and higher rewards than those with low-quality relationships (low LMX) (Fletcher and Brannick, 2022). Afshan et al. (2022) assert that “followers experiencing high-quality exchange relationships feel capable and worthy and thus are found to reciprocate by showing positive attitudes, greater psychological contract fulfillment, excellent performance, lower turnover intentions, and higher citizenship behaviors” (p. 238). This emphasizes that perceived fairness and quality of the relationship are crucial in determining how employees respond behaviorally.

LMX theory also suggests that leaders establish trust, respect, and appreciation relationships with some employees more than others, which impacts job perceptions and performance. Recent LMX theory highlights the development of close, dyadic relationships between leaders and their subordinates. Leaders favor employees with whom they share strong connections, offering them significant roles and informal mentoring. These relationships can build mutual trust and respect (Graen and Uhl-Bien, 1995). High LMX positively correlates with task performance and OCB (Martin et al., 2016). In contrast, destructive leadership negatively impacts employee perceptions of LMX and their OCB and task performance (Mackey et al., 2021).

One important factor in LMX is how employees interpret their leaders’ intentions and behavior. Even when a leader behaves in a way that seems helpful, employees may see it as manipulative, especially if the leader shows Dark Triad (DT) traits. These leaders often break social norms of fairness and respect (Elsawy et al., 2022; LeBreton et al., 2018; Moorman et al., 2025), which hurts trust and the sense of fairness that underpins LMX.

From a resource-based view, employee perception of LMX is a crucial factor in resource availability (Liang et al., 2022). Therefore, when employees perceive the relationship as instrumental or deceptive, LMX may be weakened. Social exchange theory provides another theoretical basis for the mediating role of LMX. According to the theory, relationships between leaders and employees are transactional, involving the exchange of resources and social capital (Cropanzano and Mitchell, 2005). DT leaders tend to exhibit manipulative agentic behaviors, use social influence strategies aimed at (mis)using others to serve their personal interests, and are less concerned with meeting social requirements such as the norm of reciprocity. By relying on the social exchange perspective, it was suggested that DT leaders tend to disrupt the social harmony and balance of reciprocity in social exchanges at work, which reduces LMX and team levels performance (Fodor et al., 2021). In addition, when employees perceive leadership as manipulative, or the misusing of norms of reciprocity, due to distrust in leadership, they may withdraw from the exchange, leading to defensive or negative behaviors (Palmer et al., 2020). These violations are often subjectively interpreted by employees, and their reactions depend not only on what leaders report but on how the leaders are perceived.

Empirical research demonstrates a robust effect, suggesting that LMX predicts both employee performance and OCB (see Martin et al., 2016, for a meta-analysis) and that LMX mediates the relationship between leadership’s reporting of DT and team performance (Fodor et al., 2021). By expanding these findings, we suggest that perceived DT leadership trait will reduce employee OCB through decreased perception of LMX. Furthermore, we expand previous work by suggesting a unique path of the mediation process for each DT dimension.

*Psychopathic* leaders often disregard social norms by bullying and impulsive, callous behavior, which can lead to a toxic work environment (Jones and Paulhus, 2010). From a resource perspective, psychopathic leadership increases job demands and depletes follower resources by exposing followers to cope with ongoing norm- and toxic behavior. As Moorman et al. (2025) explain, “working for a psychopathic leader means knowing that they cause harm, yet, due to their charm and charisma, followers are likely to be surprised when it occurs” (p. 4668). In line with this, they note that “followers will face increased job demands and will deplete resources because they must respond to psychopathic leaders who test and breach the boundaries established by social norms.”

Narcissistic leaders often exploit available resources primarily to strengthen their own reputation and maintain a sense of superiority in the workplace (Resick et al., 2009). This self-centered orientation frequently displays abusive supervision, where leaders utilize hostile verbal and non-verbal behaviors, such as humiliation and social undermining, to establish control (Nevicka et al., 2018). In some cases, this exploitation extends to taking credit for employees’ work (Chen et al., 2018). Thus, because these leaders are perceived as both unsupportive and exploitative (Fehn and Schütz, 2021; Tokarev et al., 2017), their behavior depletes followers’ cognitive, psychological, and emotional resources, eventually fuelling workplace hostility (Chen et al., 2024).

*Machiavellian* leaders may foster mistrust and manipulation among followers, damaging harmonious relationships (Dahling et al., 2009; Palmer et al., 2020). These leaders may increase followers’ job demands because working for them requires managing the leader’s propensity to manipulate the relationship to gain advantages. Trying to protect themselves from the leader’s self-serving actions can drain employees’ energy and lead to emotional exhaustion and even withdrawal (Moorman et al., 2025).

These mechanisms may also be relevant for management. Palmer et al. (2020) proposed a theoretical model suggesting that CEO Dark Triad traits influence the quality of exchanges with top management team (TMT) members and how these relationships cascade down to affect other employees. Specifically, they argued that *narcissistic* CEOs would reduce the exchange of quality relationships with their managers because of their self-focus and self-aggrandizing behaviors. *Machiavellian* CEOs tend to engage in manipulative, authoritarian, controlling, and overtly political behaviors, which, over time, negatively impact the quality of their exchanges with team members. Finally, *psychopathic* CEOs tend to lack empathy for others and engage in callous and abusive behaviors, which also negatively affect the exchange quality with their employees over time.

Building on Palmer et al.'s (2020) theoretical framework, we propose that LMX similarly mediates the relationship between perceived leader Dark Triad traits and employee outcomes such as OCB in our organizational context. As such, we propose the following hypothesis:

*H2*: Leadership Machiavellianism, narcissism, and psychopathy decrease employee OCB through the mediating role of LMX in both the managerial and non-managerial samples such as:

*H2a*: Leadership Machiavellianism decreases employee OCB through the mediating role of LMX in both the managerial and non-managerial samples.

*H2b*: Leadership narcissism decreases employee OCB through the mediating role of LMX in both the managerial and non-managerial samples.

*H2c*: Leadership Psychopathy decreases employee OCB through the mediating role of LMX in both the managerial and non-managerial samples.

## Political skill mediates the relationship between leadership DT and OCB

Political behavior in organizations has drawn the attention of scholars and researchers since the early 80’s. This facet of organizational life has been regarded as one of the major determinants of employees’ attitudes and behaviors and a central key to understanding organizational dynamics and consequences. This line of inquiry, however, is relatively controversial (for review, see Ferris et al., 2019). While some theoretical work (e.g., Mintzberg, 1983) refers to actions aimed at acquiring, developing, and using power and other resources to achieve desired outcomes as relatively *negative* phenomena, others view politics more neutrally (e.g., Pfeffer, 1983). In our work, political skill, defined as the ability to effectively understand others at work and use that knowledge to influence them to act in ways that enhance one’s personal or organizational objectives, is often regarded as a critical leadership competency (Ahearn et al., 2004; Kimura, 2015).

Research has shown that politically skilled leaders can develop and maintain cognitive and relational social capital with essential others to acquire and leverage critical resources, predict follower organizational commitment (Treadway et al., 2004), and increase firm performance (Ahearn et al., 2004; Kimura, 2015). In addition, politically skilled leaders can establish a strong social network that fosters relationships in the workplace (Ferris et al., 2019), increases trust, and positively shapes employee behavior, including OCB (Li and Kong, 2015; Riaz et al., 2018). However, the employment of political skill has disadvantages. For example, leaders high in political skill can conceal their self-serving behaviors (Treadway et al., 2007) or even misuse their skills to manipulate, exploit, or bully subordinates (Treadway et al., 2013).

Importantly, much of the existing literature has relied on leaders’ self-reports of political skill, which may not fully capture how those behaviors are perceived by others. Meurs et al. (2010) argue that while self-ratings reflect internal confidence, followers’ assessments more accurately reflect the leader’s interpersonal behavior and influence. Thus, the perception of the leader’s political skill, not merely the leader’s self-perceived skill, is a meaningful predictor of employees’ reactions, including their motivation to engage in OCB. In other words, even if leaders consider themselves politically skilled, it is ultimately the employees’ subjective perception that determines whether that skill is experienced as strategic social competence or manipulative influence.

This distinction is particularly important when contemplating leaders high in DT traits. For instance, Narcissistic leaders may display political charm but come across to employees as self-centered or exploitative (Krasikova et al., 2013). We theorize that Machiavellians may use calculated political moves that appear misleading or threatening. Moreover, Psychopathic leaders, lacking empathy, may not even attempt to mask their manipulation. Therefore, followers may perceive these behaviors as lacking sincerity, which in turn diminishes OCB.

Political skill has been suggested as a potential pathway to explain why individuals who possess darker personality traits may be able to advance more easily in their careers (Kückelhaus and Blickle, 2024; Lainidi et al., 2023). Previous findings indicate that leaders high in DT use their personal abilities in a self-serving and manipulative manner to pursue their own personal goals (Bueno-de la Fuente et al., 2025; Haar and de Jong, 2023). For example, Haar and de Jong (2023) argued that CEOs with Dark Triad traits adopt an agentic and exploitative social strategy, using impression management tactics to enhance external performance indicators (e.g., breakthrough sales), while internally, subordinates experience manipulation, entitlement, and a decline in managerial capital.

According to the Conservation of Resources (COR) theory, employees who face resource-draining conditions at work tend to “enter a defensive mode to preserve the self that is often aggressive and may become irrational” (Hobfoll et al., 2018, p. 104). Indeed, workplace incivility depletes employees’ relational and emotional resources, prompting them to withdraw from extra-role behaviors, such as OCB (Gümüştaş and Karataş Gümüştaş, 2023). Such behaviors, triggered by DT leaders, can create resource-draining situations (Tokunbo and Borisade, 2025), which encourage employees to adopt negative work behaviors, such as decreased OCB.

Based on this rationale, we suggest that DT leaders may actively use political skills. However, rather than employing it to build trust or foster collaboration, we argue that these leaders may use political behaviors aimed primarily at achieving their own goals. Therefore, employees who work under Machiavellian, narcissistic, and psychopathic leaders may be exposed to more self-serving and manipulative aspects of political skill, which may ultimately decrease their motivation to engage in OCB.

However, we propose that the mediating effect of political skill may not be uniform across the DT traits. While we expect the perceived political skill of psychopathic leaders to mediate the relationship between psychopathy and OCB negatively, the case of narcissistic and Machiavellian leaders is more ambiguous. Narcissistic and Machiavellian leaders are often perceived as socially competent and persuasive (Ferris et al., 2019), which may lead employees to view their political behavior as more legitimate or even beneficial in some cases. Therefore, it remains unclear whether non-managerial employees’ perception of their political skill will decrease OCB.

Machiavellians and narcissists may effectively utilize political skills to achieve workplace status. In contrast, individuals high in psychopathy may be perceived as having lower political skills, weaker reputations, and weaker workplace status. These negative perceptions stem from their tendencies toward meanness and poor impulse control, which often overshadow any strengths, such as boldness (Kückelhaus and Blickle, 2024). This distinction highlights the importance of examining each trait separately in relation to how political skill is perceived and its impact on OCB. In light of these insights, we propose the following hypotheses:

*H3*: Leadership Machiavellianism, narcissism, and psychopathy are related to employee OCB through the mediating role of Leaders’ Perceived Political Skill in both the managerial and non-managerial samples, such that:

*H3a*: Leadership Machiavellianism is related to employee OCB through the mediating role of Leaders’ Perceived Political Skill in both the managerial and non-managerial samples

*H3b*: Leadership narcissism is related to employee OCB through the mediating role of Leaders’ Perceived Political Skill in both the managerial and non-managerial samples

*H3c*: Leadership Psychopathy is related to employee OCB through the mediating role of Leaders’ Perceived Political Skill in both the managerial and non-managerial samples

## Materials and methods

### Participants and procedure in both samples

Sample Ι refers to non-managerial employees, while Sample ΙΙ refers to middle-level managers. Although both groups were asked to assess their direct supervisor, this distinction reflects the hierarchical differences relevant to our hypotheses. Throughout the manuscript, we refer to these groups as the non-managerial sample and the managerial sample, respectively, to ensure clarity in our comparative analysis.

Sample Ι consisted of 378 non-managerial employees, all of whom had been employed for at least 1 year. This group included 57.4% females and 42.6% males. Mean age was 41.6 years (SD = 11.24). 44.7% held a B.A. degree, and 18.8% held an M.A. degree. The average tenure in the organization was 8.67 years (SD = 6.65). Sample 2 included 340 managers who had been employed for at least 1 year. This group comprised 60.3% females and 39.7% males. Mean age was 44.06 years (SD = 11.51). 47.6% held a B.A. degree, and 25.9% held an M.A. degree. The average tenure in this group was 10.53 years (SD = 8.0).

Data was collected using the Prolific platform, and all questionnaires were administered and distributed via Qualtrics. Because recruitment was based on employment status criteria (non-managerial for Sample 1 and managers for Sample 2), the participants represented a broad spectrum of industries and organizations (e.g., healthcare, finance, technology, and education).

Participants were required to have at least 1 year of tenure with their current organization to ensure sufficient familiarity with their direct supervisor. Following the operationalization of “long-term relationships” in previous research (Hung et al., 2025), the current researchers defined relationships between leaders and followers as those lasting at least 1 year with regular interaction. Such duration emphasizes that sufficient time enables a meaningful exchange and an accurate perception of direct leadership.

### Measurements

Dark Triad traits and political skill were assessed through participants’ subjective evaluations of their immediate supervisor (managers for the non-managerial sample and senior executives for the managerial sample). In contrast, OCB was measured via self-reports, reflecting how participants see their own behavior at work. LMX was also assessed from the participants’ perspective to capture their subjective experience of the dyadic relationship. We consistently referred to the direct supervisor to ensure that all external evaluations focused strictly on the direct leader and the appropriate hierarchical level.

#### Leader’s Dark Triad

The Dark Triad traits of the supervisor were assessed using a 12-item scale (Jonason and Webster, 2010), adapted for use in a supervisor evaluation format. Participants were instructed to “please indicate, on a scale between 1 and 6, the extent to which you agree/disagree with each of the following statements about your immediate boss/supervisor at your present job.” The scale comprises three distinct subscales, each consisting of four items: *Machiavellianism*, which measures the leader’s use of deceit and manipulation (e.g., “*My leader tends to exploit others towards his/her own end”; α* = 0.91 for non-managerial sample; α = 0.92 for the managerial sample); *Psychopathy*, which assesses a lack of remorse and moral concern (e.g., *“My leader tends to not be too concerned with morality or the morality of his/her actions”*; α = 0.93 for non-managerial sample; α = 0.94 for managerial sample); and *narcissism*, which evaluates the leader’s need for admiration and status (e.g., *“My leader tends to want others to admire him/her”*; α = 0.92 for non-managerial sample; α = 0.94 for managerial sample). Higher scores reflect a stronger presence of these dark personality factors in the perceived leadership behavior.

#### Organizational citizenship behavior (OCB)

OCB was assessed using the 10-item Organizational Citizenship Behavior Checklist (OCB-C) (Fox et al., 2011; Spector et al., 2010; Spector, 2013). This scale was selected because it was designed to minimize overlap with counterproductive work behaviors (Spector et al., 2010). Participants indicated the frequency of various citizenship behaviors on a 6-point scale (1 = *Never* to 6 = *Every day*).

While the main analyses utilized the overall OCB score (computed by summing all 10 items), the OCB-C also allows for the assessment of two distinct dimensions: acts directed toward individuals (OCBP; similar to OCBI) and acts directed toward the organization (OCBO) (Pupunden and Susanto, 2023; Spector, 2013). OCBP comprises 5 items reflecting behaviors aimed at helping coworkers (e.g.*, “Helped a co-worker who had too much to do,” “Lent a compassionate ear when someone at work had a work problem”*). OCBO comprises 5 items reflecting behaviors benefiting the organization (e.g., *“Offered suggestions to improve how work is done,” “Volunteered for extra work assignments”*). The scale demonstrated good reliability across both samples. For the overall construct: α = 0.898 (non-managerial) and α = 0.894 (managerial). For the sub-scales, reliability was also satisfactory: OCBP (α = 0.867 for non-managers; α = 0.872 for managers) and OCBO (α = 0.831 for non-managers; α = 0.837 for managers).

#### LMX

The quality of Leader-Member Exchange relationships with the immediate supervisor was measured using Scandura and Graen’s (1984) seven-item measurement, assessed on a 6-point Likert scale (e.g., *“I have enough confidence in him/her that I would defend and justify his/her decision if he/she were not present to do so”*). (α = 0.78 for the non-managerial sample; α = 0.77 for the managerial sample).

*Perception of the Leader’s political skill* was measured using a 17-item Political Skill Inventory (PSI) (Ferris et al., 2005). The participants responded to items assessing four theoretically distinct dimensions: networking ability, apparent sincerity, social astuteness, and interpersonal influence. The participants rated their immediate supervisor. Therefore, for non-managerial employees, this typically referred to a manager, while for managers, it referred to their direct supervisor from senior management. The participants were asked to assess their superior on a 1 (*strongly disagree*) to 6 (*strongly agree*) Likert point scale (“*He/she is good at using his/her connections and network to make things happen at work”*). Cronbach’s α for this scale was high (α = 0.96 for the non-managerial sample; α = 0.96 for the managerial sample).

## Results

### Preliminary analysis and descriptive statistics

A confirmatory factor analysis (CFA) using the Structural Equation Model was performed before testing the hypothesized model. The CFA consisted of the following research variables: Machiavellianism (4 items), narcissism (4 items), psychopathy (4 items), LMX (7 items), OCB (10 items), and Leader political skill (17 items). The measurement model for the non-managerial sample showed an acceptable fit with the data (*χ*^2^(953) = 2065.74, *p* = <0.001, *χ*^2^/*df* = 2.168, CFI = 0.933, TLI = 0.927, RMSEA = 0.056. SRMSEA = 0.058); All items loaded more than 0.50 in their latent factor. The employee single-factor measurement model showed a poor fit with the data for the non-managerial sample (*χ*^2^(965) = 5965.344, *p* < 0.001, *χ*^2^/*df* = 6.175, CFI = 0.699, TLI = 0.677, RMSEA = 0.117, SRMSEA = 0.124). In addition, the measurement model demonstrated an acceptable fit with the data for the managerial sample (*χ*^2^(951) = 2177.13, *p* < 0.001, *χ*^2^/*df* = 2.289, CFI = 0.917, TLI = 0.910, RMSEA = 0.062; SRMSEA = 0.067). All items loaded more than 0.46 in their latent factor. The single-factor measurement model for the measurement scale exhibited a poor fit with the data for the managerial sample (*χ*^2^(966) = 6691.45, *p* < 0.001, *χ*^2^/*df* = 6.927, CFI = 0.613, TLI = 0.586, RMSEA = 0.132, SRMSEA = 0.154). For structural equation modeling (SEM), latent variables were constructed for each of the study constructs. For most variables (Machiavellianism, narcissism, psychopathy, LMX, and OCB), latent factors were based directly on their individual items. However, to improve model parsimony while preserving construct validity, political skill was modeled using four item parcels, each representing one of the theoretical dimensions (social astuteness, interpersonal influence, networking ability, and apparent sincerity), following the method that captures each key dimension separately (Little et al., 2002).

Descriptive statistics and correlation matrix are presented for the non-managerial sample in Table 1, and for the managerial sample in Table 2. In both samples, all three DT leadership traits were negatively associated with LMX and leader political skill. In addition, in both samples, leader political skill and LMX were positively related to OCB (at both managerial and non-managerial levels).

**Table 1:** Descriptive statistics and correlations among study variables for the non-managerial sample.

Variable	Mean	SD	1	2	3	4	5	6
1. Leader Machiavellianism	2.39	1.20	(0.91)					
2. Leader psychopathy	2.29	1.24	0.83^***^	(0.93)				
3. Leader narcissism	2.82	1.36	0.78^***^	0.70^***^	(0.92)			
4. LMX	4.30	1.17	−0.60^***^	−0.65^***^	−0.51^***^	(0.78)		
5. Leader political skill	4.34	0.96	−0.52^***^	−0.64^***^	−0.42^***^	0.76^***^	(0.96)	
6. OCB	27.42	8.50	−0.02	−0.08	−0.01	0.20^***^	0.16^**^	(0.89)

*N* = 378, ^*^p < 0.05, ^**^p < 0.01, ^***^p < 0.001. Mach, Machiavellianism; Cronbach’s alpha reliabilities are presented on the diagonal. All variables were normally distributed.

**Table 2:** Descriptive statistics and correlations among study variables for the managerial sample.

Variable	Mean	SD	1	2	3	4	5	6
1. Leader Machiavellianism	2.64	1.29	(0.93)					
2. Leader psychopathy	2.47	1.35	0.84^***^	(0.94)				
3. Leader narcissism	3.14	1.37	0.72^***^	0.67^***^	(0.94)			
4. Leader LMX	4.52	1.00	−0.45^***^	−0.56^***^	−0.37^***^	(0.77)		
5. Leader political skill	4.42	0.89	−0.33^***^	−0.50^***^	−0.27^***^	0.72^***^	(0.96)	
6. Manager OCB	32.06	8.43	0.04	0.01	0.06	0.22^***^	0.31^***^	(0.90)

*N* = 340, ^*^p < 0.05, ^**^p < 0.01, ^***^p < 0.001. Alpha Machiavellianism: Cronbach’s alpha reliabilities are presented on the diagonal.

Finally, across both samples (non-managerial employees and managers), the zero-order correlations between leader Dark Triad traits (Machiavellianism, narcissism, and psychopathy) and subordinate OCB were not statistically significant. We continued with the mediation analyses, based on research suggesting that a significant total or direct effect is not necessary to establish mediation (Edwards and Lambert, 2007; Hayes, 2018; O’Rourke and MacKinnon, 2015; Zhao et al., 2010). The mediation analysis mainly focuses on the significance of the indirect effect, which may exist even when the total effect is non-significant. Accordingly, we conducted mediation analyses for both samples to examine whether LMX and perceived political skill mediate the relationships between perceived leader Dark Triad traits and employee OCB.

### Non-managerial model testing

**Figure 1:**
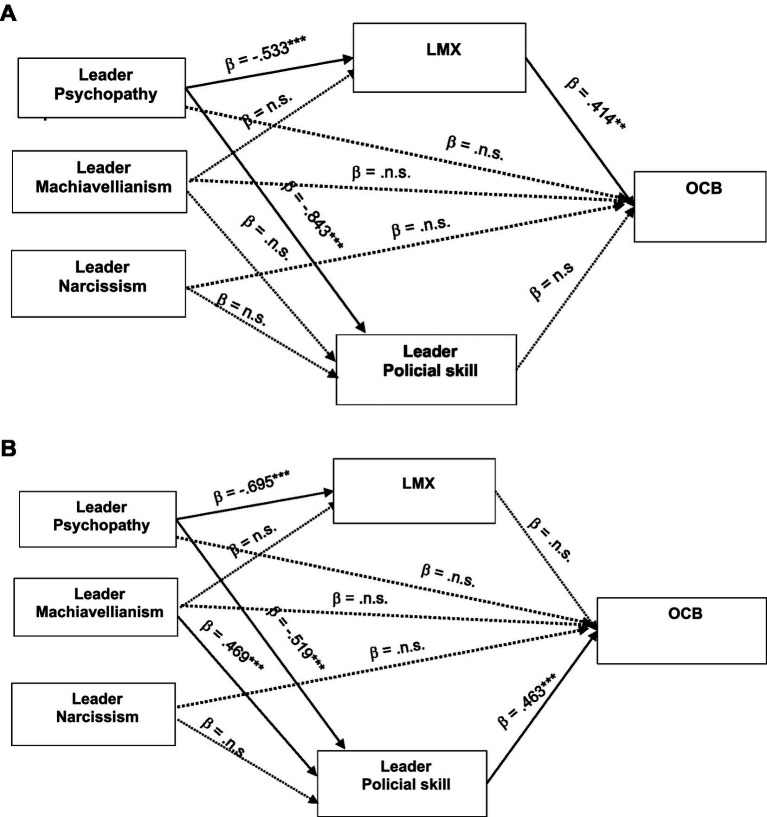
**(a)** Mediating role of political skill and LMX in the relationship between leaders’ dark triad traits and OCB (non-managerial sample). **(b)** Mediating role of political skill and LMX in the relationship between leaders’ dark triad traits and OCB (managerial sample).

SEM was used to examine the study’s research hypotheses. The results of the hypothesized model provided good model fit for the non-managerial sample [*χ*^2^(439) = 947.44; *χ*^2^/*df* = 2.158 *p* < 0.001; NFI = 0.918; TLI = 0.948; CFI = 0.954; SRMR = 0.063; RMSEA = 0.055]. H1a, H1b, and H1c were not supported; the direct relationships between leadership traits (Machiavellianism, psychopathy, and narcissism) and OCB at the non-managerial sample were not significant (*β* = 0.354, *p* = 0.164; *β* = −0.210, *p* = 0.353; *β* = −0.001, *p* = 0.970). The SEM results are shown in Table 3 and Figure 1a.

**Table 3:** SEM bootstrapping (95% CI) for the standardized indirect effects for the non-managerial sample.

Variable		Path			Coef.	LL	UL	Sig.
Leader Machiavellianism	→	LMX			−0.218	−0.564	0.148	0.223
Leader psychopathy	→	LMX			−0.533	−0.823	−0.214	<0.001
Leader narcissism	→	LMX			0.017	−0.146	0.165	0.834
Leader Machiavellianism	→	Political skill			0.094	−0.246	0.517	0.606
Leader psychopathy	→	Political skill			−0.843	−0.962	−0.562	<0.001
Leader narcissism	→	Political skill			0.057	−0.107	0.209	0.499
LMX	→	OCB			0.414	0.150	0.671	0.002
Leader political skill	→	OCB			−0.088	−0.369	0.182	0.541
Leader Machiavellianism	→	OCB			0.354	−0.148	0.864	0.164
Leader psychopathy	→	OCB			−0.210	−0.658	0.234	0.353
Leader narcissism	→	OCB			0.001	−0.153	0.153	0.970
Leader Machiavellianism	→	LMX	→	OCB	−0.063	−0.229	0.030	0.159
Leader Machiavellianism	→	Political skill	→	OCB	−0.006	−0.121	0.018	0.403
Leader psychopathy	→	LMX	→	OCB	−0.145	−0.313	−0.050	0.001
Leader psychopathy	→	Political skill	→	OCB	0.049	−0.104	0.221	0.511
Leader narcissism	→	LMX	→	OCB	0.005	−0.040	0.008	0.390
Leader narcissism	→	Political skill	→	OCB	−0.003	−0.043	0.051	0.790

The bias correction bootstrap technique using the confidence interval method was applied to examine the mediation hypotheses. Partial support for H2 was found; the indirect effect of leader psychopathy on non-managerial OCB through LMX was significant (indirect effect = −0.145, *p* = 0.001, 95% CI = [−0.313, −0.050]), thus supporting H2c. However, the indirect effects of leadership Machiavellianism and narcissism on OCB, via LMX, were not statistically significant. Hence, H2a and H2b were not supported. Regarding H3, in the non-managerial sample, the hypothesized mediation of leaders’ political skill between leadership Dark Triad traits and OCB was not statistically significant. Thus, H3a, H3b and H3c were not supported for the non-managerial sample.

### Managerial sample: model testing

SEM was used to examine the study’s research hypotheses. The results of the hypothesized model demonstrated good fit for the managerial sample [*χ*^2^(470) = 1174.453; *χ*^2^/df = 2.499, *p* < 0.001; NFI = 0.892; TLI = 0.924; CFI = 0.932; SRMR = 0.0584; RMSEA = 0.066]. H1a, H2b, and H2c were not supported. The direct relationship between Leadership dark traits (Machiavellianism, psychopathy, and narcissism) and managerial OCB was non-significant (*β* = −0.139, *p* = 0.453; *β* = 0.197, *p* = 0.109; *β* = 0.130, *p* = 0.134). The SEM results are shown in Table 4 and Figure 1b.

**Table 4:** SEM bootstrapping (95% CI) for the standardized indirect effects for the managerial sample.

Variable		Path			Coef.	LL	UL	Sig.
Leader Machiavellianism	→	LMX			0.096	−0.217	0.390	0.524
Leader psychopathy	→	LMX			−0.695	−0.960	−0.398	<0.001
Leader narcissism	→	LMX			0.011	−0.148	0.169	0.879
Leader Machiavellianism	→	Political skill			0.469	0.161	0.799	<0.001
Leader psychopathy	→	Political skill			−0.519	−0.696	−0.371	<0.001
Leader narcissism	→	Political skill			0.013	−0.153	0.172	0.861
LMX	→	OCB			0.059	−0.178	0.285	0.602
Leader political skill	→	OCB			0.463	0.220	0.685	<0.001
Leader Machiavellianism	→	OCB			−0.139	−0.602	0.254	0.453
Leader psychopathy	→	OCB			0.197	−0.070	0.417	0.109
Leader narcissism	→	OCB			0.130	−0.046	0.306	0.134
Leader Machiavellianism	→	LMX	→	OCB	0.003	−0.012	0.050	0.490
Leader Machiavellianism	→	Political skill	→	OCB	0.119	0.037	0.249	0.001
Leader psychopathy	→	LMX	→	OCB	−0.021	−0.120	0.054	0.583
Leader psychopathy	→	Political skill	→	OCB	−0.240	−0.402	−0.118	<0.001
Leader narcissism	→	LMX	→	OCB	0.001	−0.009	0.017	0.784
Leader narcissism	→	Political skill	→	OCB	0.003	−0.039	0.046	0.837

To test the mediating effects, standardized indirect effects were examined using bootstrapping (5,000 resamples). Hypothesis 3 predicted that perceptions of leaders’ political skill would mediate the relationship between the three Dark Triad traits and managerial OCB. Results provided only partial support for the hypothesis. Specifically, leader psychopathy had a significant negative indirect effect on managerial OCB through leaders’ political skill (*β* = −0.240, *p* < 0.001, 95% CI [−0.402, −0.118]), thereby supporting H3c. Machiavellianism exhibited a significant positive indirect effect on managerial OCB through the mediating role of leaders’ political skill (*β* = 0.119, *p* < 0.001, 95% CI [0.037, 0.249]), thus supporting H3a. However, political skill did not mediate the relationship between narcissism and OCB; therefore, H3b was not supported. In addition, no significant indirect effects through LMX were found for any of the predictors, indicating that H2a, H2b, and H2c were not supported for the managerial sample.

The main analyses were conducted using the overall OCB score derived from the 10-item OCB Checklist (Spector et al., 2010). To examine whether the findings held across dimensions of organizational citizenship behavior, supplementary SEM models were estimated separately for citizenship behaviors directed toward individuals (OCBP) and toward the organization (OCBO). The pattern of results, including the direction and significance of the paths, was identical to that obtained in the main model.

## Discussion

This study examined how perceived leader Dark Triad traits relate to follower OCB across hierarchical levels, exploring LMX and perceived political skill as mediators. First, we examined the relationship between perceived leaders’ DT traits and OCB across non-managerial and managerial samples. Second, while H1a, H1b, and H1c—that predicted direct negative relationships between perceived leadership DT traits and OCB—were not supported, we found consistent indirect effects that offer deeper insight into the underlying mechanisms. Specifically, in the non-managerial sample, only H2c was supported, indicating that perceived leader psychopathy was associated with reduced OCB through lower-quality leader–member exchange (LMX). In the managerial sample, support was found for H3a and H3c, though in opposite directions. Specifically, Machiavellian leadership was associated with higher OCB through more favorable perceptions of the leader’s political skill (H3a), whereas psychopathic leadership was associated with lower OCB through reduced perceived political skill (H3c). These findings indicate that the indirect effects of DT traits on OCB operate through distinct mediating mechanisms depending on employee role: LMX for non-managers and perceived political skill for managers. This demonstrates that the impact of perceived dark leadership is role-specific and context-dependent.

Though the direct effects were not significant between dark traits (narcissism, Machiavellianism, and psychopathy) and OCB, significant indirect effects were found, consistent with past research findings (Raineri and Cartes, 2024; Utomo and Siahaan, 2023). These findings indicate that the influence of dark leadership traits is not straightforward but rather unfolds through specific psychological and relational processes. In both samples, psychopathy was indirectly associated with reduced OCB but via different mediators: low-quality LMX among non-managerial employees, and reduced perceptions of leaders’ political skill among the managerial sample. Thus, H2c was supported for the non-managerial sample, while H3c was supported for the managerial sample. In addition, Machiavellian leadership showed a significant indirect effect on OCB via perceptions of political skill, but only among managers (H3a). Overall, our findings reflect the study’s differentiated approach, highlighting the unique mechanisms by which each Dark Triad trait influences follower behavior. The results support viewing these traits as separate constructs that operate through different pathways, shaped by organizational context and hierarchical level.

Our findings are consistent with the Conservation of Resources (COR) theory (Hobfoll et al., 2018) and align with prior evidence (e.g., Gümüştaş and Karataş Gümüştaş, 2023), showing that destructive leadership perceptions can deplete employees’ relational and emotional resources, resulting in withdrawal from OCB. Specifically, we identified distinct pathways of resource depletion depending on the employee’s hierarchical level. Among non-managerial employees, low LMX reflects a decrease in relational resources, which in turn reduces their willingness to engage in OCB. In contrast, among managers, we found that the decrease in leadership political competence signals a loss of strategic resources needed to navigate complex organizational environments. These differentiated pathways suggest that followers interpret and respond to dark leadership traits through distinct psychological mechanisms, depending on their position within the organizational hierarchy. By comparing these two groups, the study reveals how the same destructive trait (e.g., psychopathic leadership) can trigger context-specific resource losses, which are relational in one case and strategic in the other, ultimately leading to a reduction in prosocial workplace behavior.

For the non-managerial sample, perception of leadership psychopathy indirectly decreased OCB through deteriorated leader-member exchange (LMX) (H2c). This finding can further support the argument that non-managerial followers may be more sensitive to LMX as their day-to-day experiences are embedded in direct, interpersonal interactions with their immediate supervisors, and they may be more sensitive to relationship quality mechanisms as they can carry significant risks for them (Lee et al., 2021; Yuan et al., 2023). Given the inherent power imbalance in leader-follower relationships, followers may be especially sensitive to ambiguity, as misinterpreting the quality of the relationship can carry significant consequences (Yuan et al., 2023). Their immediate leader often serves as the primary gatekeeper to resources, information, opportunities, and organizational rewards (Yuan et al., 2023). Consequently, LMX becomes vital to their work experience and their willingness to engage in extra-role behaviors (for reviews, see Ilies et al., 2007; Yuan et al., 2023). It also serves as a potential mediator between abusive supervision and OCB among subordinates (Xu et al., 2012).

Our research findings suggest that psychopathic leadership is significantly associated with lower levels of LMX, particularly among non-managerial employees. This implies that employees perceive psychopathic leaders as less capable of building high-quality relationships. These findings are consistent with prior research which has shown that psychopathic leaders, due to their emotional coldness, impulsivity, and interpersonal antagonism, tend to undermine effective workplace relationships (Bueno-de la Fuente et al., 2025). In addition, psychopathic leaders tend to dismiss or ignore employee feedback, thereby damaging the quality of LMX with their employees (Palmer et al., 2020). Given their generally low investment in social relationships, psychopaths exhibit less concern about the potential negative consequences of their hostile behavior (O’Boyle et al., 2013; Priesemuth and Bigelow, 2020). Psychopaths often lack the social skills necessary to understand the thoughts and feelings of their colleagues, making it difficult for them to establish the social networks that support healthy workplace relationships (Cesinger et al., 2023). Based on COR theory assumptions (Hobfoll et al., 2018), when non-managerial employees perceive their leader as emotionally unavailable or exploitative, the decision to withdraw OCB becomes a protective response, a conservation strategy in the face of depleted social and emotional resources.

In contrast, LMX did not emerge as a significant mediator for the managerial sample. Instead, the managers’ perceptions of their senior leader’s political skill played a central role. Supporting H3c, when managers perceived their senior leader as psychopathic, this was associated with negative perceptions of their leaders’ political skill, which in turn reduced their OCB. For participants in the managerial sample, perceived psychopathy in their senior leader was associated with lower assessments of the leader’s political skill, a key interpersonal resource for navigating complex organizational environments (Kimura, 2015), which, in turn, predicted reduced OCB among this group. Based on COR theory (Hobfoll et al., 2018), this decrease in perceived political competence represents a depletion of strategic resources necessary for effective organizational navigation. A psychopathic leader, characterized by impulsivity and poor social judgment (Moorman et al., 2025), may be perceived as politically incompetent, unable to form effective partnerships and demonstrate the social intelligence necessary for influencing others. For managers, this perceived incompetence signals that their senior leader cannot provide the required political support or strategic positioning to enable them to succeed in their own roles. This may diminish perceptions of leadership’s social resources and ultimately impair managers’ willingness to engage in OCB.

Conversely, when the leader was perceived as Machiavellian, managers attributed higher political skill to them, which increased managerial OCB. Our results enable more nuanced assessments of leadership effectiveness based on political competence rather than purely relational qualities. This pattern aligns with findings suggesting that Machiavellian leaders may possess political skills (Palmer et al., 2020) and are more adept at transforming political skill into desired social success. Moreover, Machiavellians may build influence by gaining access to organizational resources (Kückelhaus and Blickle, 2024). These resources may be the reason for our findings regarding the increased OCB among managers. When managers perceive their senior leader as strategically competent, able to navigate organizational politics and secure resources, they may increase their OCB as a form of strategic investment.

These findings may be further explained by the inherently political nature of managerial work itself. Managers occupy a unique structural position. They are simultaneously subordinates to senior leadership and leaders of their own employees. This dual role requires them to navigate both upward and downward influence, balance strategic direction with operational demands (Taylor and Helfat, 2009), while remaining particularly sensitive to ambiguity, relational risk, and political signaling (Ellis, 2025). In this context, the heightened importance of leaders’ political skill for managers’ OCB becomes more understandable. A politically skilled senior leader can secure resources, build coalitions, and create opportunities that benefit both managers and their teams. Therefore, a senior leader perceived as lacking political skill may be seen as a liability, someone who cannot effectively advocate for the unit or navigate organizational complexity on behalf of subordinates.

Our findings align with prior research suggesting that managers are more attuned to organizational politics than employees at other levels (Lee et al., 2021). They participate in higher-level meetings, have visibility into cross-functional dynamics, and interpret leadership behavior through a strategic lens, viewing political skill as critical for navigating organizational demands (Moraes et al., 2023; Snell et al., 2014). As indicated, this broader scope enables managers to judge leadership effectiveness based on political competence rather than purely relational qualities. Thus, managers who operate in politically complex environments place greater value on their leaders’ political skill when deciding whether to engage in discretionary behaviors such as OCB. For them, the question is not primarily “Does my leader care about me?” but rather “Can my leader effectively navigate the organization to secure advantages for our unit?”. This interpretation helps explain why, in our findings, perceived political skill emerged as a more salient predictor of OCB among managers than among non-managerial employees.

To effectively drive organizational dynamics forward, managers increasingly need political skills to communicate and implement persuasive tactics (Mahajan and Templer, 2021). In this context, political skill serves as a valuable resource (Chen et al., 2021), which may explain why Machiavellian leaders, who are particularly adept at transforming political skill into social success (Blickle et al., 2020; Kückelhaus and Blickle, 2024), are sometimes perceived as functional when aligned with these organizational role demands (Moorman et al., 2025). Therefore, lower perceived political skill in psychopathic leaders serves as a critical mediator for reduced OCB (H3c), while Machiavellian leaders increase OCB through political skill (H3a) among managers.

Taken together, these findings challenge trait-centric models of leadership and underscore that the same dark trait can yield different mediating pathways across hierarchical positions. The results necessitate a more dynamic, process-oriented approach to understanding dark leadership, one that considers hierarchical differences, relational dynamics, and the complex interplay between leader characteristics and follower perceptions. Ultimately, this research demonstrates that leadership effectiveness is shaped not by traits alone, but by how those traits are interpreted and experienced across different organizational contexts.

### Practical implications

This study offers several practical implications for organizations seeking to improve employee OCB. A central contribution of the current research is its demonstration that the impact of leaders’ DT traits on OCB depends not only on the traits themselves, but also on the social mechanisms through which employees interpret and experience them. By demonstrating that psychopathic and Machiavellian leaders exert their influence primarily through indirect mechanisms, the study highlights the importance of monitoring relational quality and perceptions of leader capability as early indicators of OCB. Accordingly, interventions should focus on improving these relational and perceptual processes. For the non-managerial sample, the results indicate that weakened LMX is the primary pathway through which psychopathic leadership reduces employee OCB. Organizations can therefore mitigate negative effects by strengthening day-to-day relational quality, encouraging open communication, and training leaders to build trust with direct reports.

In contrast, among managers, psychopathy was linked to lower perceptions of political skill, while Machiavellianism was linked to higher political skill, which in turn led to more OCB. This means that when Machiavellian traits are perceived as signs of political skill, they may encourage managers to exceed their formal duties. Understanding these differences can help HR practitioners tailor interventions, anticipate risks, and design differentiated development programs that reflect the unique pressures employees and managers face. For instance, while relational interventions may be most effective for protecting non-managerial employees from toxic leadership, strategic and political competence training may better serve managers.

Furthermore, the study demonstrates that Machiavellianism and psychopathy have distinct practical implications, especially at different hierarchical levels. This suggests that organizations should avoid one-size-fits-all approaches to addressing DT leadership. Instead, they may adopt a more nuanced perspective that considers both the leader’s specific traits and the organizational hierarchy.

### Limitations and recommendations for future research

This study has several limitations. First, its cross-sectional design limits the ability to draw causal conclusions. Second, the use of employee reports may introduce bias. To reduce common method bias, we combined self-reported OCB with observer-based ratings of leaders’ DT traits and political skill. However, because each participant rated all variables, the use of a single source within each sample remains a limitation. Future studies should consider adopting multi-source designs, in which data are collected from different organizational roles (e.g., subordinates and supervisors), to reduce common method bias. Additionally, longitudinal research designs are recommended to better assess causal relationships over time. Additionally, because we did not include behavioral measures, our findings are limited to perceptions rather than actual behaviors.

Despite these limitations, the study offers several strengths. It is grounded in theory and applies a two-sample design that includes both non-managerial and managerial samples. Additionally, we employed structural equation modeling (SEM) with adequate sample sizes (*n* = 378 and *n* = 340) to test complex mediation models. This design offers a broader understanding of how DT personality traits function across organizational levels. Finally, participants were recruited via an online academic panel (Prolific), resulting in a multi-industry sample rather than data from a specific organization or sector. While this diversity increases the generalizability of the results across various work settings, it also means that organizational-specific variables (such as industry norms or corporate culture) were not controlled.

Future research should address the limitations of this study by employing longitudinal designs and incorporating behavioral measures to establish causal relationships, thereby gaining a more nuanced understanding of the impact of DT traits on workplace outcomes. Further investigation could also delve into the potentially adaptive aspects of DT traits in different organizational and cultural contexts. For example, certain leadership dark triad traits may be perceived as functional or even desirable in high-stakes or performance-driven environments, such as military, medical, and show business milieu. In these contexts, qualities such as assertiveness, risk-taking, or strategic manipulation might be viewed as useful or necessary for success. In addition, future research may explore how meta-perceptions (individuals’ beliefs about how others perceive them) interact with leaders’ dark personality traits to shape employee outcomes, such as OCB. This mechanism may explain why dark traits lead to adaptive behaviors in some contexts but not others, particularly across hierarchical levels. Finally, to gain a clearer understanding of the nonsignificant direct relationship between perceived Dark Triad leadership and follower OCB, future studies should explore potential moderators, such as follower personality traits or organizational role (e.g., marketing or operations) that might shape this association.

## Conclusion

This study examined the relationship between perceived leaders’ Dark Triad traits and OCB across two hierarchical levels. Results showed that psychopathy was negatively related to followers’ OCB in both samples, but through different mediators: lower LMX among non-managerial employees and lower perceived political skill among managers. Machiavellianism was positively associated with OCB among managers, primarily due to enhanced perceptions of political skill. These findings highlight the importance of considering mediating mechanisms and hierarchical context when assessing the impact of dark leadership traits on OCB.

## Data Availability

The authors will make the raw data supporting the conclusions of this article available upon request, without undue reservation.
